# Tumor size may influence the prognosis of solitary hepatocellular carcinoma patients with cirrhosis and without macrovascular invasion after hepatectomy

**DOI:** 10.1038/s41598-021-95835-5

**Published:** 2021-08-11

**Authors:** Bin-yong Liang, Jin Gu, Min Xiong, Er-lei Zhang, Zun-yi Zhang, Xiao-ping Chen, Zhi-yong Huang

**Affiliations:** 1 Hepatic Surgery Center, Tongji Hospital, Tongji Medical College, Huazhong University of Science and Technology, 1095 Jie Fang Da Dao, Wuhan, 430030 China; 2 Department of Hepatobiliary and Pancreatic Surgery, The Second Affiliated Hospital, School of Medicine, Zhejiang University, Hangzhou, China; 3 Department of Gynecology, Tongji Hospital, Tongji Medical College, Huazhong University of Science and Technology, Wuhan, China

**Keywords:** Liver cancer, Hepatocellular carcinoma

## Abstract

Hepatocellular carcinoma (HCC) is usually associated with varying degrees of cirrhosis. Among cirrhotic patients with solitary HCC in the absence of macro-vascular invasion, whether tumor size drives prognosis or not after hepatectomy remains unknown. This study aimed to investigate the prognostic impact of tumor size on long-term outcomes after hepatectomy for solitary HCC patients with cirrhosis and without macrovascular invasion. A total of 813 cirrhotic patients who underwent curative hepatectomy for solitary HCC and without macrovascular invasion between 2001 and 2014 were retrospectively studied. We set 5 cm as the tumor cut-off value. Propensity score matching (PSM) was performed to minimize the influence of potential confounders including cirrhotic severity that was histologically assessed according to the Laennec staging system. Recurrence-free survival (RFS) and overall survival (OS) were compared between the two groups before and after PSM. Overall, 464 patients had tumor size ≤ 5 cm, and 349 had tumor size > 5 cm. The 5-year RFS and OS rates were 38.3% and 61.5% in the  ≤ 5 cm group, compared with 25.1% and 59.9% in the > 5 cm group. Long-term survival outcomes were significantly worse as tumor size increased. Multivariate analysis indicated that tumor size > 5 cm was an independent risk factor for tumor recurrence and long-term survival. These results were further confirmed in the PSM cohort of 235 pairs of patients. In cirrhotic patients with solitary HCC and without macrovascular invasion, tumor size may significantly affect the prognosis after curative hepatectomy.

## Introduction

Hepatocellular carcinoma (HCC) is the fifth most common cancer and the third leading cause of cancer-related mortality in the world^1,2^. Currently, hepatectomy remains the mainstay curative treatment for HCC patients^3^. Improvements in surgical techniques and perioperative management have improved the safety of hepatectomy. However, due to the high HCC recurrence rate, the long-term outcome of HCC patients after curative hepatectomy remains unsatisfactory^4^.

Patients with large HCC are known to have worse prognosis than those with small HCC after curative hepatectomy^5,6^. This is often because large HCC is more frequently correlated with other adverse clinicopathological factors influencing tumor recurrence and long-term survival, such as multiplicity, satellite nodules, macrovascular invasion, or distant metastasis^7,8^. However, in patients with solirary HCC and without macrovascular invasion, the relationship between tumor size and long-term outcomes after curative hepatectomy remains controversial.

Studies have shown that most HCC patients were associated with hepatitis-related cirrhosis^9,10^. Cirrhosis is not a consistent entity in terms of histological changes and can be further divided from mild to severe according to the fibrous septal thickness and nodule size^11^. Histological severity of cirrhosis has been validated to be useful in predicting prognosis in HCC patients who underwent hepatectomy with curative intent^12,13^. Therefore, evaluating the influence of tumor size on prognosis needs adjustment for confounding factor in the background liver, including histological severity of cirrhosis.

In this study, we aimed to elucidate the prognostic impact of tumor size on long-term outcomes in cirrhotic patients with solitary HCC and without macrovascular invasion after curative hepatectomy.

## Materials and methods

### Patients

A consecutive series of 813 patients with solitary HCC and without macrovascular invasion who underwent curative hepatectomy at Tongji Hospital between 2001 and 2014 were included in this study. All patients were associated with histologically diagnosed cirrhosis. Clinicopathological data of all patients were obtained from the computerized database maintained in our hospital. Portal hypertension was defined as the presence of either esophageal varices on endoscopy or splenomegaly with a platelet count < 100 × 10^9^/L^14^. Cirrhotic severity was histologically staged according to the Laennec staging system as follows: F4A, mild cirrhosis, definite or probable; F4B, moderate cirrhosis (at least 2 broad septa); and F4C, severe cirrhosis (at least 1 very broad septum or many minute nodules)^11^. A major hepatectomy was defined as resection of ≥ 3 Couinaud liver segments^15^.This study was approved by the ethics committee of Tongji Hospital, Huazhong University of Science and Technology, China. Written informed consent was obtained from each patient in the study for his/her data to be used in clinical research. All methods were carried out in accordance with relevant guidelines and regulations.

### Follow-up

All patients were evaluated by serum alpha-fetoprotein (AFP), ultrasonography or computed tomography, and chest X-ray 1 month after surgery. Patients were then followed-up once every 2 months for the first 2 years and once every 3 months thereafter. Further magnetic resonance imaging, bone scans, or positron emission tomography were performed if tumor recurrence was suspected. Patients with tumor recurrence were actively treated with repeat resection, microwave or radiofrequency ablation, ethanol injection, transarterial chemoembolization (TACE), radiotherapy, or oral sorafenib depending on the general condition of the patients, HCC recurrence pattern, and liver functional status. Overall survival (OS) was calculated from the date of hepatectomy to the date of either death or last follow-up. Recurrence-free survival (RFS) was calculated from the date of hepatectomy to the date of recurrence or death or last follow-up.

### Statistical analysis

Categorical variables were reported as number (n) and proportion (%) and compared using Pearson’s χ^2^ analysis. Continuous variables were reported as median and range. The RFS and OS were calculated using the Kaplan–Meier method and compared using the log-rank test. The Cox proportional hazards model was used to identify independent risk factors associated with RFS and OS by multivariate analysis. A *P* value < 0.05 was set as the significance threshold.

To balance the background risks between the two study groups, we performed 1:1 propensity score matching (PSM) using a caliper of 0.1 and to include age, gender, etiology, alanine aminotransferase, portal hypertension, Child–Pugh grade, AFP, extent of hepatectomy, intraoperative blood transfusion, histological severity of cirrhosis, microvascular invasion, and tumor differentiation. The PSM model was generated using the PSM program through the SPSS R-Plugin. The analysis applied single nearest-neighbor matching.

For all tests, a 2-tailed *P* < 0.05 was considered statistically significant. Statistical analysis was performed using the SPSS 26 statistical software (SPSS, Inc., Chicago, IL, USA).

## Results

### Baseline characteristics

The characteristics of patients are shown in Table 1. Among the 813 patients enrolled in the present study, 717 patients (88.2%) were male, and 96 (11.8%) were female. Seven hundred and sixty-four patients (94.0%) were with Child–Pugh grade A liver function, and 49 (6.0%) were with Child–Pugh grade B liver function. The majority etiology of HCC was hepatitis B, accounting for 91.6% of the entire cohort. The median tumor diameter was 4.7 cm (range 1.0–20.0 cm). Four hundred and sixty-four patients (57.1%) had tumor size ≤ 5 cm, and 349 (42.9%) had tumor size > 5 cm. Minor hepatectomy was performed for 713 patients (87.7%), and major hepatectomy was performed for 100 patients (12.3%). According to the Laennec staging system, 376 patients (46.2%) were diagnosed with mild cirrhosis, 360 (44.3%) with moderate cirrhosis, and 77 (9.5%) with severe cirrhosis. One hundred and thirty-eight patients (17.0%) were associated with microvacular invasion.

**Table 1:** Baseline characteristics of the entire cohort.

Characteristics	Value
Age, median (range), years	49 (16–82)
**Gender, n (%)**	
Male	717 (88.2%)
Female	96 (11.8%)
**Etiology, n (%)**	
Hepatitis B	745 (91.6%)
Hepatitis C	6 (0.7%)
Other	62 (7.6%)
Alanine aminotransferase, median (range), U/L	36.0 (5.0–381)
Total bilirubin, median (range), μmol/L	13.3 (2.0–172.9)
Albumin, median (range), g/L	40.1 (22.3–53.6)
PT, median (range), s	12.1 (9.2–18.1)
Platelet count, median (range), 10^9^/L	132 (16–484)
**Portal hypertension, n (%)**	
Absent	579 (71.2%)
Present	234 (28.8%)
**Child–Pugh grade, n (%)**	
A	764 (94.0%)
B	49 (6.0%)
**AFP, n (%), ng/mL**	
≤ 400	521 (64.1%)
> 400	292 (35.9%)
Tumor size, median (range), cm	4.7 (1.0–20.0)
**Tumor size, n (%), cm**	
≤ 5	464 (57.1%)
> 5	349 (42.9%)
**Extent of hepatectomy, n (%)**	
Minor hepatectomy	713 (87.7%)
Major hepatectomy	100 (12.3%)
Intraoperative blood transfusion, n (%)	151 (18.6%)
**Histological severity of cirrhosis, n (%)**	
Mild cirrhosis	376 (46.2%)
Moderate cirrhosis	360 (44.3%)
Severe cirrhosis	77 (9.5%)
**Microvascular invasion, n (%)**	
Absent	675 (83.0%)
Present	138 (17.0%)
**Tumor differentiation, n (%)**	
Well	179 (22.0%)
Moderate	436 (53.6%)
Poor	198 (24.4%)

*PT* prothrombin time, *AFP* alpha-fetoprotein.

The PSM cohort comprised 470 patients, gouped into 235 with tumor size ≤ 5 cm and 235 with tumor size > 5 cm. The characteristics between the ≤ 5 cm and > 5 cm groups before and after PSM are shown in Table 2. Before PSM, the proportions of patients with portal hypertension and moderate/severe cirrhosis were lower in the > 5 cm group than in the ≤ 5 cm group. However, the proportions of patients with AFP > 400 ng/mL, microvascular invasion, and moderate/poor tumor differentiation were higher in the > 5 cm group than in the ≤ 5 cm group. Besides, compared with those in the > 5 cm group, patients in the ≤ 5 cm group had a higher level of alanine aminotransferase. After PSM, there was no significant difference in clinicopathological features between the two matched groups (all *P* > 0.05).

**Table 2:** Clinicopathological characteristics by tumor size and propensity score matching.

Variables	Before PSM			After PSM		
	≤ 5 cm (n = 464)	> 5 cm (n = 349)	*P*	≤ 5 cm (n = 235)	> 5 cm (n = 235)	*P*
**Age, n (%), years**			0.169			0.486
≤ 60	391 (84.3%)	306 (87.7%)		203 (86.4%)	208 (88.5%)	
> 60	73 (15.7%)	43 (12.3%)		32 (13.6%)	27 (11.5%)	
**Gender, n (%)**			0.694			0.393
Male	411 (88.6%)	306 (87.7%)		210 (89.4%)	204 (86.8%)	
Female	53 (11.4%)	43 (12.3%)		25 (10.6%)	31 (13.2%)	
**Etiology, n (%)**			0.366			0.338
Hepatitis B/C	432 (93.1%)	319 (91.4%)		218 (92.8%)	223 (94.9%)	
Other	32 (6.9%)	30 (8.6%)		17 (7.2%)	12 (5.1%)	
**Alanine aminotransferase, n (%), U/L**			0.004			0.512
≤ 40	310 (66.8%)	199 (57.0%)		134 (57.0%)	141 (60.0%)	
> 40	154 (33.2%)	150 (43.0%)		101 (43.0%)	94 (40.0%)	
**Portal hypertension, n (%)**			< 0.001			0.754
Absent	308 (66.4%)	271 (77.7%)		171 (72.8%)	174 (74.0%)	
Present	156 (33.6%)	78 (22.3%)		64 (27.2%)	61 (26.0%)	
**Child–Pugh grade, n (%)**			0.757			0.805
A	435 (93.8%)	329 (94.3%)		227 (96.6%)	226 (96.2%)	
B	29 (6.2%)	20 (5.7%)		8 (3.4%)	9 (3.8%)	
**AFP, n (%), ng/mL**			< 0.001			0.851
≤ 400	328 (70.7%)	193 (55.3%)		138 (58.7%)	140 (59.6%)	
> 400	136 (29.3%)	156 (44.7%)		97 (41.3%)	95 (40.4%)	
**Extent of hepatectomy, n (%)**			< 0.001			1.000
Minor hepatectomy	459 (98.9%)	254 (72.8%)		230 (97.9%)	230 (97.9%)	
Major hepatectomy	5 (1.1%)	95 (27.2%)		5 (2.1%)	5 (2.1%)	
Intraoperative blood transfusion, n (%)	45 (9.7%)	106 (30.4%)	< 0.001	38 (16.2%)	39 (16.6%)	0.901
**Histological severity of cirrhosis, n (%)**			0.027			0.116
Mild cirrhosis	199 (42.9%)	177 (50.7%)		35 (14.9%)	48 (20.4%)	
Moderate/severe cirrhosis	265 (57.1%)	172 (49.3)		200 (85.1%)	187 (79.6%)	
**Microvascular invasion, n (%)**			0.001			0.636
Absent	403 (86.9%)	272 (77.9%)		193 (82.1%)	189 (80.4%)	
Present	61 (13.1%)	77 (22.1%)		42 (17.9%)	46 (19.6%)	
**Tumor differentiation, n (%)**			0.002			0.116
Well	120 (25.9%)	59 (16.9%)		35 (14.9%)	48 (20.4%)	
Moderate/poor	344 (74.1%)	290 (83.1%)		200 (85.1%)	187 (79.6%)	

*PSM* propensity score matching, *AFP* alpha-fetoprotein.

### Survival outcomes

During a median follow-up of 44.0 months, 484 patients (59.5%) suffered from HCC recurrence, and 280 patients (34.4%) died. The 1-, 3-, 5-, 7-, and 10-year RFS and OS rates of the entire cohort were 65.4%, 45.6%, 32.6%, 26.9%, and 21.1%, respectively, and 88.7%, 72.1%, 60.3%, 45.5%, and 35.6%, respectively.

**Figure 1:**
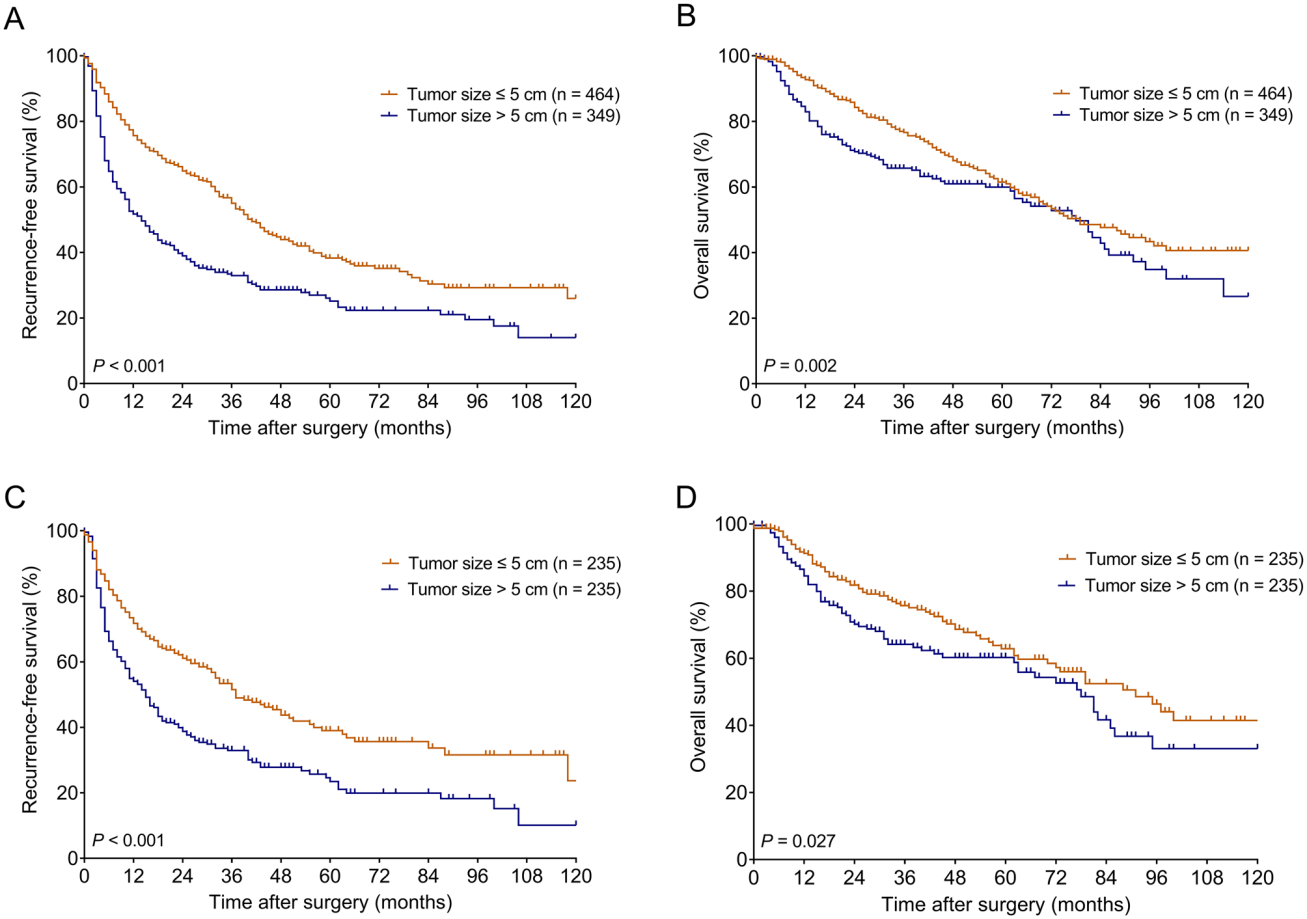
Kaplan–Meier survival curves in the groups stratified by tumor size in the entire cohort (**A**,**B**) and in the PSM cohort (**C**,**D**). (**A**,**C**) RFS curves. (**B**,**D**) OS curves. *PSM* propensity score matching, *RFS* recurrence-free survival, *OS* overall survival.

Before PSM, the 1-, 3-, 5-, 7, and 10-year RFS rates were 75.7%, 55.0%, 38.3%, 30.3%, and 26.0%, respectively, in patients with tumor size ≤ 5 cm, and 51.7%, 39.0%, 25.1%, 22.3%, and 14.1%, respectively, in patients with tumor size > 5 cm (Fig. 1A). Patients in the > 5 cm group had worse RFS than those in the ≤ 5 cm group (*P* < 0.001). The 1-, 3-, 5-, 7-, and 10-year OS rates were 92.7%, 76.6%, 61.5%, 47.7%, and 40.6%, respectively, in patients with tumor size ≤ 5 cm, and 82.9%, 65.7%, 59.9%, 42.8%, and 26.6%, respectively, in patients with tumor size > 5 cm (Fig. 1 B). Patients in the > 5 cm group had worse OS than those in the ≤ 5 cm group (*P* = 0.002).

After PSM, the 1-, 3-, 5-, 7, and 10-year RFS rates were 71.7%, 51.5%, 39.0%, 33.7%, and 23.7%, respectively, in patients with tumor size ≤ 5 cm, and 54.0%, 32.9%, 23.4%, 19.9%, and 10.1%, respectively, in patients with tumor size > 5 cm (Fig. 1C). Patients in the > 5 cm group had worse RFS than those in the ≤ 5 cm group (*P* < 0.001). The 1-, 3-, 5-, 7-, and 10-year OS rates were 91.2%, 75.7%, 62.8%, 52.5%, and 41.4%, respectively, in patients with tumor size ≤ 5 cm, and 84.5%, 64.1%, 60.2%, 41.6%, and 33.1%, respectively, in patients with tumor size > 5 cm (Fig. 1D). Patients in the > 5 cm group had worse OS than those in the ≤ 5 cm group (*P* = 0.027).

**Figure 2:**
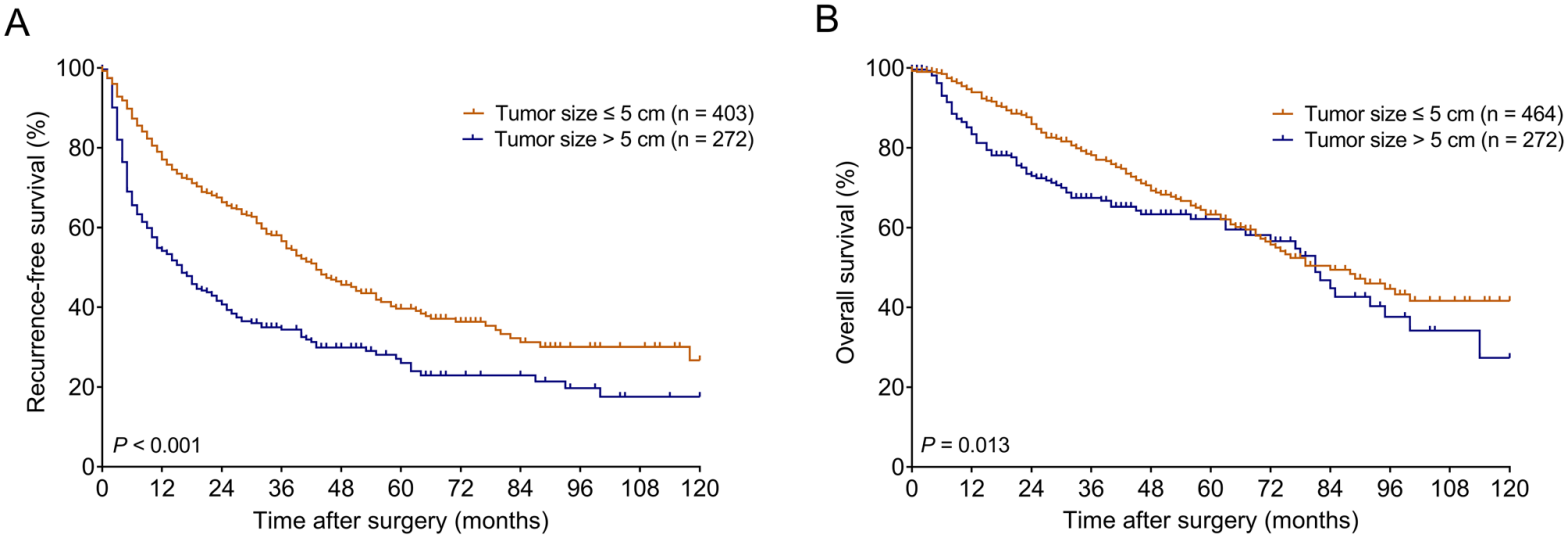
Kaplan–Meier survival curves in the groups stratified by tumor size in patients without microvascular invasion. (**A**) RFS curves. (**B**) OS curves. *RFS* recurrence-free survival, *OS* overall survival.

Further analysis was performed in patients without microvascular invasion. Among these patients, the 1-, 3-, 5-, 7, and 10-year RFS rates in the ≤ 5 cm and > 5 cm groups were 77.0%, 56.6%, 39.7%, 31.2%, and 26.7%, and 54.1%, 34.4%, 27.1%, 22.9%, and 17.5%, respectively (Fig. 2A); while the 1-, 3-, 5-, 7, and 10-year OS rates in the ≤ 5 cm and > 5 cm groups were 93.9%, 78.1%, 63.3%, 49.4%, and 41.7%, and 83.4%, 67.5%, 62.1%, 44.8%, and 27.3%, respectively (Fig. 2B). Patients in the > 5 cm group had worse RFS (*P* < 0.001) and OS (*P* = 0.013) than those in the ≤ 5 cm group.

### Factors associated with recurrence-free and overall survival

Univariate analysis showed that presence of hepatitis B/C virus infection, portal hypertension, Child–Pugh grade B liver function, AFP > 400 ng/mL, tumor size > 5 cm, major hepatectomy, intraoperative blood transfusion, moderate/severe cirrhosis, microvascular invasion, and moderate/poor tumor differentiation were associated with worse RFS (Table 3). Furthermore, portal hypertension, Child–Pugh grade B liver function, AFP > 400 ng/mL, tumor size > 5 cm, intraoperative blood transfusion, moderate/severe cirrhosis, microvascular invasion, and moderate/poor tumor differentiation were associated with worse OS (Table 4). These significant prognostic variables identified by the univariate analysis were entered into the Cox proportional hazards model for multivariate analysis, which confirmed portal hypertension, AFP > 400 ng/mL, tumor size > 5 cm, moderate/severe cirrhosis, and moderate/poor tumor differentiation as independent adverse prognostic factors for RFS (Table 5). Furthermore, portal hypertension, AFP > 400 ng/mL, tumor size > 5 cm, moderate/severe cirrhosis, microvascular invasion, and moderate/poor tumor differentiation were identified as independent adverse prognostic factors for OS (Table 5).

**Table 3:** Univariate analysis of clinicopathological variables associated with recurrence-free survival.

Variable	Before PSM			After PSM		
	n	5-year RFS (%)	*P*	n	5-year RFS (%)	*P*
**Age, years**			0.440			0.756
≤ 60	697	32.7		411	31.9	
> 60	116	31.2		59	26.6	
**Gender**			0.158			0.375
Male	717	31.7		414	30.7	
Female	96	38.4		56	35.5	
**Etiology**			0.017			0.025
Hepatitis B/C	751	31.0		441	29.6	
Other	62	49.8		29	53.4	
**Alanine aminotransferase, U/L**			0.086			0.311
≤ 40	509	33.4		275	32.3	
> 40	304	31.0		195	29.9	
**Portal hypertension**			< 0.001			< 0.001
Absent	579	41.5		345	40.3	
Present	234	10.9		125	5.1	
**Child–Pugh grade**			0.018			0.019
A	764	34.1		453	32.3	
B	49	11.1		17	8.8	
**AFP, ng/mL**			< 0.001			0.002
≤ 400	521	37.0		278	35.4	
> 400	292	24.6		192	25.2	
**Tumor size, cm**			< 0.001			< 0.001
≤ 5	464	38.3		235	39.0	
> 5	349	25.1		235	23.4	
**Extent of hepatectomy**			0.003			0.478
Minor hepatectomy	713	33.1		460	31.4	
Major hepatectomy	100	32.4		10	–	
**Intraoperative blood transfusion**			< 0.001			0.001
No	662	34.5		393	33.3	
Yes	151	24.1		77	20.2	
**Histological severity of cirrhosis**			< 0.001			< 0.001
Mild cirrhosis	376	46.0		230	45.5	
Moderate/severe cirrhosis	437	18.4		240	15.2	
**Microvascular invasion**			0.001			0.060
Absent	675	34.2		382	32.6	
Present	138	24.4		88	26.3	
**Tumor differentiation**			< 0.001			0.002
Well	179	49.4		83	49.4	
Moderate/poor	634	27.6		387	27.4	

*PSM* propensity score matching, *RFS* recurrence-free survival, *AFP* alpha-fetoprotein.

**Table 4:** Univariate analysis of clinicopathological variables associated with overall survival.

Variable	Before PSM			After PSM		
	n	5-year OS (%)	*P*	n	5-year OS (%)	*P*
**Age, years**			0.285			0.261
≤ 60	697	61.3		411	62.6	
> 60	116	53.8		59	52.9	
**Gender**			0.907			0.874
Male	717	60.7		414	61.2	
Female	96	57.2		56	61.8	
**Etiology**			0.380			0.288
Hepatitis B/C	751	59.7		441	60.8	
Other	62	66.1		29	67.7	
**Alanine aminotransferase, U/L**			0.678			0.724
≤ 40	509	59.3		275	59.3	
> 40	304	61.4		195	63.6	
**Portal hypertension**			< 0.001			< 0.001
Absent	579	68.2		345	69.9	
Present	234	40.8		125	37.4	
**Child–Pugh grade**			< 0.001			0.001
A	764	60.8		453	62.4	
B	49	51.0		17	37.6	
**AFP, ng/mL**			< 0.001			0.031
≤ 400	521	65.8		278	67.1	
> 400	292	50.1		192	52.7	
**Tumor size, cm**			0.002			0.027
≤ 5	464	61.5		235	62.8	
> 5	349	59.9		235	60.2	
**Extent of hepatectomy**			0.081			0.067
Minor hepatectomy	713	60.6		460	61.7	
Major hepatectomy	100	60.2		10	31.1	
**Intraoperative blood transfusion**			0.001			0.012
No	662	62.4		393	63.7	
Yes	151	50.2		77	47.9	
**Histological severity of cirrhosis**			< 0.001			< 0.001
Mild cirrhosis	376	73.0		230	73.4	
Moderate/severe cirrhosis	437	48.1		240	49.4	
**Microvascular invasion**			< 0.001			0.028
Absent	675	62.3		382	63.8	
Present	138	48.6		88	48.2	
**Tumor differentiation**			< 0.001			< 0.001
Well	179	76.9		83	79.1	
Moderate/poor	634	55.2		387	57.5	

*PSM* propensity score matching, *OS* overall survival, *AFP* alpha-fetoprotein.

**Table 5:** Independent prognostic factors for recurrence-free and overall survival by multivariate analysis.

Variable	Before PSM			After PSM		
	HR	95% CI	*P*	HR	95% CI	*P*
**Recurrence-free survival**						
Portal hypertension						
Absent	Ref	–	–	Ref	–	–
Present	1.626	1.311–2.016	< 0.001	1.622	1.214–2.166	0.001
AFP, ng/mL						
≤ 400	Ref	–	–	Ref	–	–
> 400	1.344	1.115–1.619	0.002	1.454	1.150–1.840	0.002
Tumor size, cm						
≤ 5	Ref	–	–	Ref	–	–
> 5	1.772	1.454–2.160	< 0.001	1.796	1.421–2.271	< 0.001
Intraoperative blood transfusion						
No	Ref	–	–	Ref	–	–
Yes	1.199	0.947–1.517	0.131	1.424	1.041–1.949	0.027
Histological severity of cirrhosis						
Mild cirrhosis	Ref	–	–	Ref	–	–
Moderate/severe cirrhosis	1.593	1.297–1.957	< 0.001	1.494	1.142–1.953	0.003
Tumor differentiation						
Well	Ref	–	–	Ref	–	–
Moderate/poor	1.602	1.259–2.038	< 0.001	1.626	1.167–2.264	0.004
**Overall survival**						
Portal hypertension						
Absent	Ref	–	–	Ref	–	–
Present	2.022	1.546–2.645	< 0.001	2.080	1.447–2.991	< 0.001
AFP, ng/mL						
≤ 400	Ref	–	–	Ref	–	–
> 400	1.405	1.102–1.791	0.006	1.536	1.120–2.106	0.008
Tumor size, cm						
≤ 5	Ref	–	–	Ref	–	–
> 5	1.486	1.154–1.914	0.002	1.563	1.146–2.133	0.005
Histological severity of cirrhosis						
Mild cirrhosis	Ref	–	–	Ref	–	–
Moderate/severe cirrhosis	1.821	1.388–2.389	< 0.001	1.717	1.194–2.468	0.004
Microvascular invasion						
Absent	Ref	–	–	Ref	–	–
Present	1.551	1.138–2.113	0.005	1.501	1.017–2.215	0.041
Tumor differentiation, n (%)						
Well	Ref	–	–	Ref	–	–
Moderate/poor	1.707	1.229–2.370	0.001	2.200	1.324–3.656	0.002

*PSM* propensity score matching, *HR* hazard ratio, *CI* confidence interval, *AFP* alpha-fetoprotein.

The factors that might affect RFS and OS after PSM were also analyzed by univariate (Table 3 and Table 4) and multivariate analysis (Table 5). Multivariate analysis further verified that tumor size > 5 cm was an independent adverse prognostic factor for both RFS and OS after PSM.

## Discussion

In this study, we analyzed the prognostic significance of clinicopathological factors especially on the tumor size for solitary HCC in cirrhotic patients without macrovascular invasion who underwent hepatectomy with curative intent. We used 5 cm as the cut-off value to classify patients into two groups and for subsequent survival analysis. Using 5 cm as the criteria has additional merit, because many studies assigned 5 cm as the cut-off value between early and intermediate stage HCC^16–19^. In addition, the cut-off value of 5 cm also was included in the Milan criteria^20^ and the Hong Kong Liver Cancer staging system^21^. The present study revealed that the size of solitary tumor was significantly correlated with the prognosis of HCC patients in the absence of macrovascular invasion after curative hepatectomy. Then, to clarify the true oncological impact of tumor size on tumor recurrence and long-term survival, we perfomed PSM analysis by adjusting for potential confounders (including tumor- and liver-related factors, especially for histological severity of cirrhosis) between the ≤ 5 cm and > 5 cm groups. We found that patients who had tumor size > 5 cm had significantly worse RFS and OS rates than their counterpart both in the entire cohort as well as in the PSM cohort.

Tumor size was a significant risk factor for tumor spread of HCC^22–24^. The frequency of intrahepatic metastasis increased by about one-third between HCC less and larger than 5 cm, and the incidence of portal vein tumor thrombosis doubled^23,24^. Previously, several studies revealed that there was a negative correlation between tumor size and prognosis in HCC patients after hepatectomy, and poor outcomes were observed for those with large tumor size^5,6,25^. However, although these studies included patients with solitary HCC, some tumors were associated with macrovascular invasion. Among all the prognostic clinicopathological factors for long-term survival, macrovascular invasion is well known to be associated with poor prognosis and a high possibility of tumor recurrence after hepatectomy or liver transplantation for HCC^26–28^. Some studies attributed the correlation between tumor size and prognosis to the association of tumor size with other more important adverse prognostic factors including tumor-related microenvironment, nutritional status, genetic background, vascular invasion, poorer differentiation, and multifocality^8,29–34^. Several studies also concluded that tumor size did not independently influence the prognosis of solitary HCC without vascular invasion^35,36^. However, recently, several large cohort studies have demonstrated the importance of tumor size as a prognostic marker for solitary HCC^37–39^. In the current study, we confirmed that there was a significant prognostic influence of tumor size on tumor recurrence and long-term survival before and after PSM. However our retrospective study was based on a moderate sample size without independent verification cohort, a meta-analysis would be warranted for further confirming this findings with subgroup analyses based on the factors that could confound this association^40–42^.

The discrepancy between our results and those of previous studies may have resulted from our inclusion of patients with solitary HCC and without macrovascular invasion, allowing for evaluation of the true prognostic risk associated with tumor size. Another reason might be that we controlled for the confounding effects of liver-related factors. The condition of underlying cirrhosis in HCC patients is one of the most important factors to decide treatment modality as well as to influence the survival outcomes. Previous studies investigating the correlation between tumor size and prognosis in HCC patients after hepatectomy all regarded cirrhosis as a one-stage condition and ignored the difference in the histological severity of cirrhosis. Mounting evidence reveals that cirrhosis is not a single disease stage^11,43,44^. Furthermore, several studies have demonstrated that histological severity of cirrhosis is very useful in predicting prognosis in HCC patients with cirrhosis after hepatectomy^12,13^. In this study, cirrhosis was histologically staged according to the Laennec staging system, and we found that cirrhotic severity was adversely correlated with long-term outcomes in patients with solitary HCC and without macrovascular invasion. Patients with moderate/severe cirrhosis had poorer prognosis than those with mild cirrhosis, consistent with previous findings^12,13^. In the present study, the proportions of patients with portal hypertension and moderate/severe cirrhosis were lower in the > 5 cm group than in the ≤ 5 cm group. Thus, controlling for the confounding effects of liver-related factors might also have influenced the results.

Among those HCC patients without macrovascular invasion, the occurrence rate of microvascular invasion was 17.0%. Patients with large tumor size had a higher incidence of microvascular invasion, consistent with previous findings^7,38^. Although tumor size and the incidence of microvascular invasion were significantly correlated, both were independent prognostic factors for tumor recurrence and long-term survival after hepatectomy. Furthermore, there was a significant prognostic influence of tumor size on tumor recurrence and long-term survival in the subgroup of patients without microvascular invasion. In addition to microvascular invasion, we found that there was a significant correlation between tumor size and differentiation. The proportion of well differentiation was significantly higher in the ≤ 5 cm group than in the > 5 cm group. In this study, 16.9% of patients with tumor size > 5 cm have well differentiation. The most plausible explanation for such a high proportion was that this study only included cirrhotic patients with solitary HCC and without macrovascular invasion.

This study has several limitations. First, this was a retrospective study taking place in a single center, thus selection biases were unavoidable. Further multicenter and prospective studies are needed to validate the results of the current study. Second, the majority of HCC patients in this study were infected by hepatitis B virus. This feature is different from patients infected by hepatitis C virus in most Western countries or Japan.

In conclusion, this study demonstrated that in cirrhotic patients with solitary HCC and without macrovascular invasion, tumor size may significantly influence tumor recurrence and long-term survival after curative hepatectomy, however the potential causality is not clear and a Mendelian randomization study is warrant to disclose the causal effects^45–47^. Stratification of these patients according to tumor size could aid in determining prognosis and developing reasonable protocols for patient management.

## References

[1] El-Serag HB, Rudolph KL (2007). Hepatocellular carcinoma: Epidemiology and molecular carcinogenesis. Gastroenterology.

[2] Akinyemiju T, Abera S, Ahmed M, Alam N, Alemayohu MA, Allen C (2017). The burden of primary liver cancer and underlying etiologies from 1990 to 2015 at the global, regional, and national level: Results from the global burden of disease study 2015. JAMA Oncol..

[3] Bruix J, Gores GJ, Mazzaferro V (2014). Hepatocellular carcinoma: Clinical frontiers and perspectives. Gut.

[4] Tung-Ping PR, Fan ST, Wong J (2000). Risk factors, prevention, and management of postoperative recurrence after resection of hepatocellular carcinoma. Ann. Surg..

[5] Poon RT, Fan ST (2004). Hepatectomy for hepatocellular carcinoma: Patient selection and postoperative outcome. Liver Transpl..

[6] Pandey D, Lee KH, Wai CT, Wagholikar G, Tan KC (2007). Long term outcome and prognostic factors for large hepatocellular carcinoma (10 cm or more) after surgical resection. Ann. Surg. Oncol..

[7] Pawlik TM, Delman KA, Vauthey JN, Nagorney DM, Ng IO, Ikai I (2005). Tumor size predicts vascular invasion and histologic grade: Implications for selection of surgical treatment for hepatocellular carcinoma. Liver Transpl..

[8] Zhang H, Yuan SX, Dai SY, Zhang JM, Huang X, Lu CD (2014). Tumor size does not independently affect long-term survival after curative resection of solitary hepatocellular carcinoma without macroscopic vascular invasion. World J. Surg..

[9] Chen XP, Wu ZD, Huang ZY, Qiu FZ (2005). Use of hepatectomy and splenectomy to treat hepatocellular carcinoma with cirrhotic hypersplenism. Br. J. Surg..

[10] EASL-ALEH Clinical Practice Guidelines (2015). Non-invasive tests for evaluation of liver disease severity and prognosis. J. Hepatol..

[11] Kim MY, Cho MY, Baik SK, Park HJ, Jeon HK, Im CK (2011). Histological subclassification of cirrhosis using the Laennec fibrosis scoring system correlates with clinical stage and grade of portal hypertension. J. Hepatol..

[12] Kim SU, Jung KS, Lee S, Park JY, Kim DY, Ahn SH (2014). Histological subclassification of cirrhosis can predict recurrence after curative resection of hepatocellular carcinoma. Liver Int..

[13] Huang ZY, Liang BY, Xiong M, Dong KS, Zhang ZY, Zhang EL (2016). Severity of cirrhosis should determine the operative modality for patients with early hepatocellular carcinoma and compensated liver function. Surgery.

[14] Santambrogio R, Kluger MD, Costa M, Belli A, Barabino M, Laurent A (2013). Hepatic resection for hepatocellular carcinoma in patients with Child–Pugh's A cirrhosis: Is clinical evidence of portal hypertension a contraindication?. HPB (Oxf.).

[15] Pol B, Campan P, Hardwigsen J, Botti G, Pons J, Le Treut YP (1999). Morbidity of major hepatic resections: A 100-case prospective study. Eur. J. Surg..

[16] El-Serag HB (2011). Hepatocellular carcinoma. N. Engl. J. Med..

[17] Torzilli G, Belghiti J, Kokudo N, Takayama T, Capussotti L, Nuzzo G (2013). A snapshot of the effective indications and results of surgery for hepatocellular carcinoma in tertiary referral centers: Is it adherent to the EASL/AASLD recommendations?: An observational study of the HCC East-West study group. Ann. Surg..

[18] Jung YK, Jung CH, Seo YS, Kim JH, Kim TH, Yoo YJ (2016). BCLC stage B is a better designation for single large hepatocellular carcinoma than BCLC stage A. J. Gastroenterol. Hepatol..

[19] Forner A, Reig M, Bruix J (2018). Hepatocellular carcinoma. Lancet.

[20] Mazzaferro V, Regalia E, Doci R, Andreola S, Pulvirenti A, Bozzetti F (1996). Liver transplantation for the treatment of small hepatocellular carcinomas in patients with cirrhosis. N. Engl. J. Med..

[21] Yau T, Tang VY, Yao TJ, Fan ST, Lo CM, Poon RT (2014). Development of Hong Kong Liver Cancer staging system with treatment stratification for patients with hepatocellular carcinoma. Gastroenterology.

[22] Liver Cancer Study Group of Japan (1989). The general rules for the clinical and pathological study of primary liver cancer. Jpn. J. Surg..

[23] Yuki K, Hirohashi S, Sakamoto M, Kanai T, Shimosato Y (1990). Growth and spread of hepatocellular carcinoma. A review of 240 consecutive autopsy cases. Cancer.

[24] Adachi E, Maeda T, Kajiyama K, Kinukawa N, Matsumata T, Sugimachi K (1996). Factors correlated with portal venous invasion by hepatocellular carcinoma: Univariate and multivariate analyses of 232 resected cases without preoperative treatments. Cancer.

[25] Lai EC, Ng IO, Ng MM, Lok AS, Tam PC, Fan ST (1990). Long-term results of resection for large hepatocellular carcinoma: A multivariate analysis of clinicopathological features. Hepatology.

[26] Izumi R, Shimizu K, Ii T, Yagi M, Matsui O, Nonomura A (1994). Prognostic factors of hepatocellular carcinoma in patients undergoing hepatic resection. Gastroenterology.

[27] Li H, Wang X, Lu X, Zhu H, Li S, Duan S, Zhao X, Zhang F, Alterovitz G, Wang F, Li Q, Tian XL, Xu M (2019). Co-expression network analysis identified hub genes critical to triglyceride and free fatty acid metabolism as key regulators of age-related vascular dysfunction in mice. Aging (Albany NY).

[28] Li B, Yuan Y, Chen G, He L, Zhang Y, Li J (2010). Application of tumor-node-metastasis staging 2002 version in locally advanced hepatocellular carcinoma: Is it predictive of surgical outcome?. BMC Cancer.

[29] Varotti G, Ramacciato G, Ercolani G, Grazi GL, Vetrone G, Cescon M (2005). Comparison between the fifth and sixth editions of the AJCC/UICC TNM staging systems for hepatocellular carcinoma: Multicentric study on 393 cirrhotic resected patients. Eur. J. Surg. Oncol..

[30] Wang X, Jiao X, Tian Y, Zhang J, Zhang Y, Li J, Yang F, Xu M, Yu X, Shanghai Birth Cohort Study (2021). Associations between maternal vitamin D status during three trimesters and cord blood 25(OH)D concentrations in newborns: A prospective Shanghai birth cohort study. Eur. J. Nutr..

[31] Yu H, Pan R, Qi Y, Zheng Z, Li J, Li H, Ying J, Xu M, Duan S (2020). LEPR hypomethylation is significantly associated with gastric cancer in males. Exp. Mol. Pathol..

[32] Jin G, Xu M, Zou M, Duan S (2020). The processing, gene regulation, biological functions, and clinical relevance of N4-acetylcytidine on RNA: A systematic review. Mol. Ther. Nucleic Acids.

[33] Zheng S, Zhao T, Yuan S, Yang L, Ding J, Cui L, Xu M (2019). Immunodeficiency promotes adaptive alterations of host gut microbiome: An observational metagenomic study in mice. Front. Microbiol..

[34] Yan X, Zhao X, Li J, He L, Xu M (2018). Effects of early-life malnutrition on neurodevelopment and neuropsychiatric disorders and the potential mechanisms. Prog. Neuropsychopharmacol. Biol. Psychiatry.

[35] Yang LY, Fang F, Ou DP, Wu W, Zeng ZJ, Wu F (2009). Solitary large hepatocellular carcinoma: A specific subtype of hepatocellular carcinoma with good outcome after hepatic resection. Ann. Surg..

[36] Ariizumi S, Kotera Y, Takahashi Y, Katagiri S, Yamamoto M (2013). Impact of hepatectomy for huge solitary hepatocellular carcinoma. J. Surg. Oncol..

[37] Chan AC, Fan ST, Poon RT, Cheung TT, Chok KS, Chan SC (2013). Evaluation of the seventh edition of the American Joint Committee on Cancer tumour-node-metastasis (TNM) staging system for patients undergoing curative resection of hepatocellular carcinoma: Implications for the development of a refined staging system. HPB (Oxford).

[38] Hwang S, Lee YJ, Kim KH, Ahn CS, Moon DB, Ha TY (2015). The impact of tumor size on long-term survival outcomes after resection of solitary hepatocellular carcinoma: Single-institution experience with 2558 patients. J. Gastrointest. Surg..

[39] Huang WJ, Jeng YM, Lai HS, Sheu FY, Lai PL, Yuan RH (2015). Tumor size is a major determinant of prognosis of resected stage I hepatocellular carcinoma. Langenbecks Arch. Surg..

[40] Jiang L, Wang K, Lo K, Zhong Y, Yang A, Fang X, Akezhuoli H, Song Z, Chen L, An P, Xu M, Min J, Wang F (2019). Sex-specific association of circulating ferritin level and risk of type 2 diabetes: A dose–response meta-analysis of prospective studies. J. Clin. Endocrinol. Metab..

[41] Chen J, Zhao X, Cui L, He G, Wang X, Wang F, Duan S, He L, Li Q, Yu X, Zhang F, Xu M (2020). Genetic regulatory subnetworks and key regulating genes in rat hippocampus perturbed by prenatal malnutrition: Implications for major brain disorders. Aging (Albany NY).

[42] Wu Y, Cao H, Baranova A, Huang H, Li S, Cai L, Rao S, Dai M, Xie M, Dou Y, Hao Q, Zhu L, Zhang X, Yao Y, Zhang F, Xu M, Wang Q (2020). Multi-trait analysis for genome-wide association study of five psychiatric disorders. Transl. Psychiatry.

[43] Gu J, Zhang E, Liang B, Zhang Z, Long X, Xiang S (2020). Use of direct liver stiffness measurement in evaluating the severity of liver cirrhosis in patients with hepatocellular carcinoma. World J. Surg..

[44] Gu J, Zhang E, Liang B, Zhang Z, Chen X, Xiong M (2021). Liver collagen contents are closely associated with the severity of cirrhosis and posthepatectomy liver failure in patients with hepatocellular carcinoma and Child–Pugh grade a liver function. Ann. Surg. Oncol..

[45] Wang X, Fang X, Zheng W, Zhou J, Song Z, Xu M, Min J, Wang F (2021). Genetic support of a causal relationship between iron status and type 2 diabetes: A Mendelian randomization study. J. Clin. Endocrinol. Metab..

[46] Zhang F, Baranova A, Zhou C, Cao H, Chen J, Zhang X, Xu M (2021). Causal influences of neuroticism on mental health and cardiovascular disease. Hum. Genet..

[47] Zhang F, Rao S, Cao H, Zhang X, Wang Q, Xu Y, Sun J, Wang C, Chen J, Xu X (2021). Genetic evidence suggests posttraumatic stress disorder as a subtype of major depressive disorder. J. Clin. Invest..

